# Stromal Vascular Fraction Therapy for Knee Osteoarthritis: A Systematic Review

**DOI:** 10.3390/medicina59122090

**Published:** 2023-11-28

**Authors:** Evgeniy Nikolaevich Goncharov, Oleg Aleksandrovich Koval, Eduard Nikolaevich Bezuglov, Manuel de Jesus Encarnacion Ramirez, Mikhail Engelgard, Eremin Ilya Igorevich, Alessandra Saporiti, Konstantin Valentinovich Kotenko, Nicola Montemurro

**Affiliations:** 1 Petrovsky Russian Scientific Center of Surgery, 121359 Moscow, Russia; 2 Department of Neurological Diseases and Neurosurgery, Peoples’ Friendship University of Russia, 121359 Moscow, Russia; 3 Department of Pharmaceuticals, Azienda Usl Toscana Nord Ovest, 56100 Pisa, Italy; 4 Department of Neurosurgery, Azienda Ospedaliero Universitaria Pisana (AOUP), 56100 Pisa, Italy

**Keywords:** knee osteoarthritis, regenerative medicine, stromal vascular fraction, personalized medicine, surgery

## Abstract

*Background and Objectives:* Knee osteoarthritis (OA) is a widespread joint disease, set to increase due to aging and rising obesity. Beyond cartilage degeneration, OA involves the entire joint, including the synovial fluid, bones, and surrounding muscles. Existing treatments, such as NSAIDs and corticosteroid injections, mainly alleviate symptoms but can have complications. Joint replacement surgeries are definitive but carry surgical risks and are not suitable for all. Stromal vascular fraction (SVF) therapy is a regenerative approach using cells from a patient’s adipose tissue. SVF addresses as degenerative and inflammatory aspects, with potential for cartilage formation and tissue regeneration. Unlike traditional treatments, SVF may reverse OA changes. Being autologous, it reduces immunogenic risks. *Materials and Methods:* A systematic search was undertaken across PubMed, Medline, and Scopus for relevant studies published from 2017 to 2023. Keywords included “SVF”, “Knee Osteoarthritis”, and “Regenerative Medicine”. *Results:* This systematic search yielded a total of 172 articles. After the removal of duplicates and an initial title and abstract screening, 94 full-text articles were assessed for eligibility. Of these, 22 studies met the inclusion criteria and were subsequently included in this review. *Conclusions:* This review of SVF therapy for knee OA suggests its potential therapeutic benefits. Most studies confirmed its safety and efficacy, and showed improved clinical outcomes and minimal adverse events. However, differences in study designs and sizes require a careful interpretation of the results. While evidence supports SVF’s positive effects, understanding methodological limitations is key. Incorporating SVF is promising, but the approach should prioritize patient safety and rigorous research.

## 1. Introduction

Knee osteoarthritis (OA) stands as one of the most prevalent and debilitating joint diseases affecting the global population. With an aging demographic and rising obesity rates, both of which are known risk factors, the incidence of knee OA is set to surge in the coming years. This chronic condition, primarily seen in the older population, inflicts significant pain, reduces joint mobility, and consequently hinders the quality of life for those affected [1,2].

The pathology of knee OA delves deeper than just the degeneration of the articular cartilage. It is a complex disorder involving the entire joint, with changes occurring in the synovial fluid, underlying bone, ligaments, and surrounding muscles. While it has traditionally been viewed as a wear-and-tear disease, modern understanding acknowledges that inflammatory processes play a crucial role in its progression [3].

Current conventional treatments, such as non-steroidal anti-inflammatory drugs (NSAIDs), corticosteroid injections, and physical therapy, predominantly focus on symptom alleviation. However, they often fall short in addressing the root causes or halting the disease’s progression. Moreover, prolonged use of such interventions can sometimes introduce additional complications, such as gastrointestinal issues with NSAIDs or cartilage degradation with repeated steroid injections [4].

Joint replacement surgeries, specifically total knee arthroplasties, offer a more definitive solution for advanced cases. However, they come with inherent risks associated with surgical procedures and are not always viable for all patients, given factors such as age, other health conditions, or personal preference. This leaves a significant therapeutic gap, highlighting the pressing need for innovative interventions that can not only manage but also potentially reverse the disease’s progression [5,6].

SVF, derived from adipose tissue, is a heterogeneous cell population which includes not only adipose-derived stem cells but also macrophages, endothelial cells, pericytes, and other cell types. The unique composition of SVF holds promise in addressing both the degenerative and inflammatory facets of knee OA. While the adipose-derived stem cells possess the potential for chondrogenesis (cartilage formation) and tissue regeneration, the other cellular components play vital roles in modulating the joint’s inflammatory environment [7].

Recent advances in regenerative medicine spotlight the capability of SVF to not only serve as a symptomatic relief option but potentially reverse some of the disease’s pathological changes. This restorative potential could position SVF as a transformative approach in knee OA management, setting it apart from conventional treatments that often focus primarily on symptom control [8].

Moreover, the autologous nature of SVF therapy—using the patient’s own cells—minimizes the risk of immunogenic reactions, offering a safer profile compared to some other therapeutic modalities. With the global prevalence of knee OA on the rise, fueled by aging populations and increasing obesity rates, innovative treatments such as SVF offer hope in changing the narrative of this debilitating condition from one of inevitable decline to potential restoration [6,9,10].

Stromal vascular fraction (SVF) therapy is a novel and promising form of regenerative medicine. SVF therapy leverages cells derived from the patient’s adipose tissue. This systematic review aims to critically evaluate the current evidence surrounding SVF therapy for knee OA:

- Addressing a Growing Global Health Issue: Knee OA is a widespread joint disease, increasingly prevalent due to aging populations and rising obesity rates. This research aims to tackle the growing burden of knee OA, which significantly affects the quality of life due to pain and reduced mobility.
- Symptom Management: Traditional treatments for knee OA, such as NSAIDs, corticosteroids, and joint replacement surgeries, often focus on symptom relief and come with various risks and limitations. In contrast, SVF therapy represents a paradigm shift towards addressing the underlying pathology of OA, offering the potential for tissue regeneration and disease modification, which could fundamentally alter the disease trajectory.
- Harnessing Regenerative Potential: The research into SVF therapy is at the forefront of regenerative medicine. SVF, derived from a patient’s own adipose tissue, contains a diverse mix of cells capable of exerting regenerative, immunomodulatory, and anti-inflammatory effects. This autologous nature minimizes the risk of immunogenic reactions, presenting a safer, personalized therapeutic option.

## 2. Materials and Methods

### 2.1. Search Strategy

A systematic search was conducted across multiple electronic databases, including PubMed, Medline and Scopus, to identify relevant studies published since 2017 until 2023. An additional hand-search of reference lists from primary articles was also executed to ensure no potential studies were missed. Keywords used were “Stromal Vascular Fraction” AND/OR “SVF” in combination with “Knee Osteoarthritis” AND/OR “Adipose-derived stem cells”, “Regenerative Medicine”, and “Intra-articular Injection”. A review protocol was entered into the PROSPERO database (ID 485428).

#### 2.1.1. Inclusion Criteria

Inclusion criteria included original research articles focused on the use of SVF therapy in knee OA, studies reporting clinical outcomes post-SVF therapy, both randomized controlled trials (RCTs) and observational studies, publications in the English language, and studies with a minimum follow-up period of three months.

#### 2.1.2. Exclusion Criteria

The following was excluded to maintain the integrity and objective of our study: studies involving animals, case reports or series with fewer than 10 subjects, reviews, meta-analyses, and non-research letters or commentaries, studies where SVF was used in combination with other regenerative therapies, making it difficult to attribute outcomes solely to SVF, research studies with a limited sample size (specifically those with fewer than 10 participants, which might not provide the robust evidence that this review aims to collate), and non-original research articles, such as commentaries, editorials, and opinion pieces.

### 2.2. Data Extraction and Quality Assessment

For each selected article, data were extracted by two independent reviewers (E.N.G. and N.M.). The data comprised the year of publication, study type, the number of participants, main findings, follow up time, and complications. Any discrepancies between the reviewers were resolved through discussion until a consensus was reached. The quality of the included studies was assessed using the Cochrane Risk of Bias tool for RCTs and the Newcastle-Ottawa Scale for observational studies. This ensured the credibility of the findings and allowed for the identification of potential biases in study methodologies.

### 2.3. Quality Assessment and Risk of Bias Analysis

#### 2.3.1. Search Strategy and Inclusion/Exclusion Criteria Strengths

The multi-database search (PubMed, Medline, and Scopus) was comprehensive and appropriate for the subject matter, ensuring a wide capture of relevant literature. The inclusion of hand-searching of reference lists reduced the risk of missing important studies. The criteria for inclusion and exclusion are clearly stated to promote transparency in the selection process.

Potential Biases/Risks: The restriction to English-language publications could introduce language bias, potentially omitting significant findings from non-English sources. The exclusion of smaller studies (less than 10 subjects) may overlook important preliminary or pilot data that could contribute to the field.

#### 2.3.2. Data Extraction and Quality Assessment Strengths

Utilizing two independent reviewers for data extraction enhances the reliability of the data gathering process. Employing established assessment tools such as the Cochrane Risk of Bias tool and the Newcastle-Ottawa Scale ensures a standardized evaluation of study quality.

Potential Biases/Risks: There is a risk of subjective interpretation during the resolution of discrepancies between reviewers, potentially leading to inconsistent data extraction or interpretation.

### 2.4. Ethical Considerations

While this systematic review involved the analysis of already published data and did not directly involve human participants, it adhered to the principles of the Declaration of Helsinki, ensuring that the studies included were ethically conducted and had necessary patient consents.

## 3. Results

**Figure 1:**
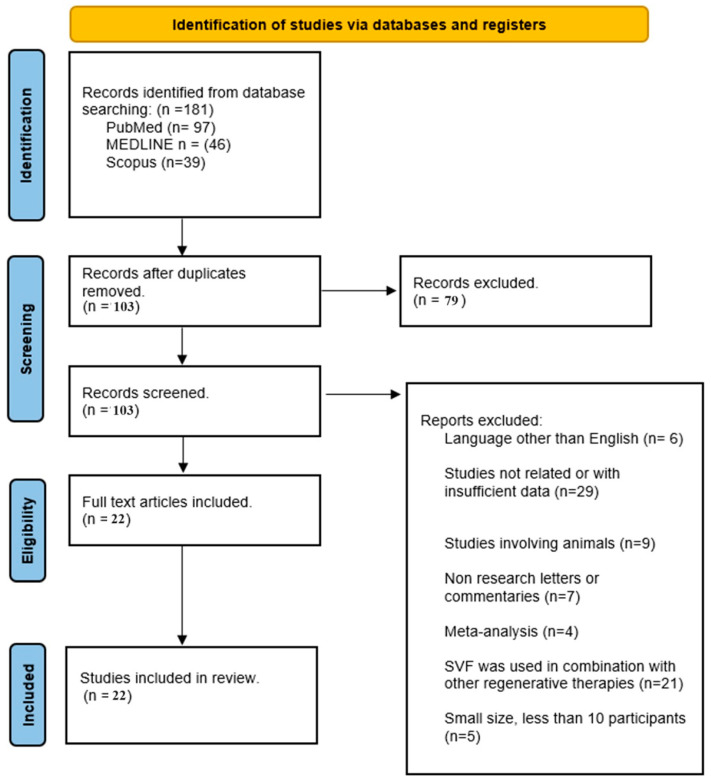
PRISMA flow diagram.

The systematic search yielded a total of 181 articles. After the removal of duplicates and an initial title and abstract screening, 104 full-text articles were assessed for eligibility. Of these, 22 studies [11,12,13,14,15,16,17,18,19,20,21,22,23,24,25,26,27,28,29,30,31,32,33] met the inclusion criteria and were subsequently included in this review (Table 1). The selected studies included prospective and/or retrospective case series, randomized controlled clinical trials, and reviews. This rigorous methodology provides a framework for a thorough and systematic analysis of the existing literature on SVF in knee OA, offering robust insights into the potential of this important cell population in regenerative medicine. We must highlight that, among the selected papers [11,12,13,14,15,16,17,18,19,20,21,22,23,24,25,26,27,28,29,30,31,32,33]. Figure 1 shows the PRISMA flow diagram.

## 4. Discussion

The therapeutic potential of SVF therapy in managing knee OA has gained significant attention in recent years. SVF, composed of a heterogeneous mixture of cells including ADSCs, pericytes, and smooth muscle cells, has shown promising outcomes in terms of regenerative, immunomodulatory, and anti-inflammatory effects (Table 2). Characterizing the purification of SVF is of paramount importance, especially when considering its application in therapeutic contexts. The purification process ensures that unwanted components, potentially harmful contaminants, or non-functional elements are removed, leaving behind a highly enriched fraction that can be safely and effectively used for regenerative purposes [34]. The purification and analysis of SVF entail a comprehensive evaluation of its cellular and molecular constituents. First, cellular composition is often deciphered using flow cytometry, which uses specific markers to quantify cell types, such as ASCs (CD34+, CD31−, and CD45−), endothelial cells (CD31+), and immune cells (CD45+). Additionally, microscopy, such as histological or fluorescent examinations, visually presents cellular composition [34,35].

The present work provided presents results from a myriad of studies that have evaluated the efficacy and safety of SVF in treating OA of the knee. A few key observations and insights from the table can be summarized as follows:

### 4.1. Efficacy and Safety

Across the board, most of the studies reported positive outcomes in multiple domains, from pain management to cartilage regeneration. Russo et al. [11] and Labarre et al. [12] emphasize the safety and feasibility of SVF therapy with no significant complications. Lapuente et al. [13], Garza et al. [14], and Yokota et al. [15] affirm that intra-articular SVF injections led to significant improvements in pain and joint function. Rothrauff et al. [16] demonstrated that augmenting the repair with a photocrosslinkable hydrogel, which contained SVF cells isolated intraoperatively through rapid enzymatic digestion, enhanced meniscal healing and reduced osteoarthritic changes. A note of optimism emerges from Kim et al. [20,21], who reported favorable cartilage regeneration in the SVF group. These findings seem promising for patients suffering from chronic knee OA and looking for alternatives to invasive surgeries (Table 3).

### 4.2. Long-Term Efficacy

An interesting dimension considered by Zhang et al. [28] is the longevity of the treatment’s effects, reported to last up to 5 years. This aspect is particularly important for a degenerative condition such as knee OA that requires long-term management. However, the latter study also notes a reduction in cartilage volume, albeit less than in the control group, over 5 years, raising questions about the ultimate durability of SVF therapy.

### 4.3. Adverse Events and Complications

A heartening trend is the limited number of adverse events across these studies. Nguyen et al. [18] was an outlier in this regard, with complications including arterial hypertension and chest pain. Importantly, this could be an artifact of the study’s design or the specific patient sample, requiring further investigation. Santoprete et al. [26] observed knee joint swelling in 7% of patients, which was transient and self-limiting.

### 4.4. Steps of SVF

**Figure 2:**
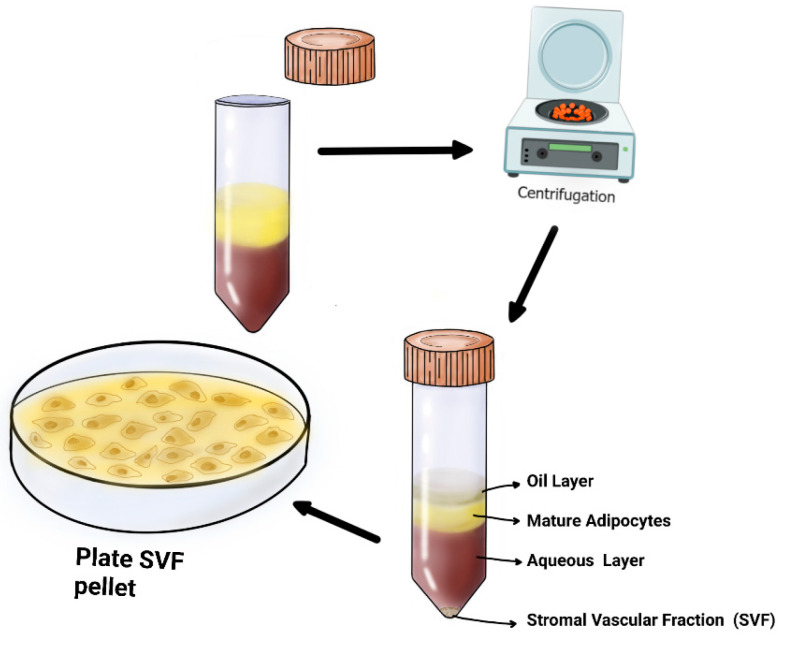
Lipoaspirate, centrifuged at 2500 or 3000 rpm for 4 min at room temperature. After centrifugation, upper oil fraction, middle condensed lipoaspirate, lower aqueous fraction, and the stromal vascular fraction were observed.

The steps of SVF separation can be summarized as (a) liposuction, (b) mechanical separation or faxination, (c) initial filtration, (d) washing, (e) final filtration, (f) SVF and adipose graft harvesting, and (g) cell counting and/or characterization (Table 4; Figure 2).

Medical devices for the preparation of AD-tSVF are summarized in Table 5 [49,50]. These devices are safely used in plastic, reconstructive, and aesthetic surgery [51]. They are also frequently used to treat Achilles tendon injuries, rotator cuff ruptures of the shoulder joint, hand flexor tendon injuries, and osteochondral defect management. Little is known, however, about their molecular action mechanism in articular cartilage [52].

### 4.5. Specificity of Response

Koh et al. [17] introduces an exciting nuance by suggesting that SVF therapy might be more effective for patients with advanced OA compared to moderate OA. If substantiated, this finding could pave the way for personalized therapy regimes in OA, tailored according to disease severity.

### 4.6. Potential for Disease Modification

Perhaps the most tantalizing hint emerging from this review is the potential for SVF therapy to not just manage but modify the course of OA. This is particularly exemplified in 2023 by Kim et al. [20] who documented cartilage regeneration, hinting at disease modification rather than mere symptom management [20]. A specific note from Aletto et al. [25] indicates that SVF therapy might be more effective in patients with more advanced OA (KL grade 3) compared to those with moderate OA (KL grade 2).

The synergy of structural and functional data, analyzed through sophisticated machine learning models, could lead to earlier and more precise interventions [72,73,74]. Such an approach is especially promising in the context of knee OA, where early detection and intervention can significantly alter the disease trajectory, potentially delaying or preventing the progression to more debilitating stages [75].

The potential of the results obtained from our research on SVF therapy for knee OA is significant and holds promise for transforming the treatment landscape of this common joint condition. Here are the key potentials of these findings:

- Innovative Treatment Approach: SVF therapy represents a novel treatment strategy, moving beyond symptom management to potentially repairing and regenerating damaged knee tissues. This approach could revolutionize the way knee OA is treated, offering a more effective solution than current methods.
- Personalized Medicine: Since SVF therapy uses cells from the patient’s own body, it aligns with the principles of personalized medicine. This individualized approach may increase the treatment’s effectiveness and reduce the risk of adverse reactions compared to standard treatments.
- Long-Term Benefits: The regenerative potential of SVF therapy suggests that its benefits could be long-lasting, potentially slowing or even halting the progression of knee OA. This long-term improvement could reduce the overall healthcare burden associated with managing chronic knee conditions.
- Safety Profile: As an autologous treatment (using the patient’s own cells), SVF therapy is expected to have a favorable safety profile with minimal risk of immune reactions. This aspect is crucial in making the treatment a viable option for a broader range of patients.

### 4.7. Limitation of this Study

While the findings from the reviewed studies are promising, several limitations must be acknowledged. The diversity in study designs, spanning from randomized controlled trials (RCTs) to retrospective observational studies and case reports series, creates challenges in comparing results and deriving consistent conclusions. The sample sizes varied considerably; some studies consisted of a single individual, while others included hundreds of participants. Understandably, studies with larger cohorts tend to offer more reliable outcomes, whereas smaller ones might not capture the broader population’s experiences. Another concern is the potential for publication bias. It is plausible that studies showcasing positive results might be favored for publication over those with less favorable or neutral outcomes, thus potentially offering a skewed representation of SVF’s effectiveness and safety. Notably, some studies, particularly case reports and observational studies, did not feature control groups. The absence of such controls complicates our ability to attribute observed benefits solely to SVF therapy.

The applicability of these findings to broader populations also raises questions. Differences in patient demographics, geographic locations of studies, and varied methodologies can impact the universal relevance of the results. Lastly, while many studies highlighted minimal to no complications, it is critical to consider that not all adverse effects, especially the milder ones, might have been exhaustively documented or reported.

## 5. Conclusions

The comprehensive review of studies on SVF therapy for knee OA has provided valuable insight into its potential therapeutic role. The majority of the studies highlighted the safety and efficacy of SVF interventions, with numerous reports of improved clinical outcomes, ranging from pain alleviation and enhanced joint mobility to encouraging signs of cartilage regeneration. Notably, adverse events associated with this therapy were largely minor or non-existent, underpinning the safety profile of SVF therapy. However, it is crucial to consider the variability in study designs, methodologies, and sample sizes when interpreting these findings. While the collective evidence leans towards the positive effects of SVF, this optimism should be balanced with an understanding of the methodological limitations inherent in the presented studies. Incorporating SVF therapy into mainstream treatment modalities for knee OA may hold significant promise. Yet, more standardized, larger-scale, and longer-term studies are essential to ascertain the definitive benefits, optimal protocols, and potential long-term risks associated with this therapy. The journey towards establishing SVF therapy as a cornerstone for knee OA management is an exciting one, but it must be undertaken with thoroughness, rigor, and an unwavering commitment to patient safety and well-being.

## Figures and Tables

**Table 1:** Studies included in this review [11,12,13,14,15,16,17,18,19,20,21,22,23,24,25,26,27,28,29,30,31,32,33].

Authors (Year)	Study Design	Sample Size	Clinical Outcomes Measured	Duration of Follow-Up	Adverse Events or Complications
Russo et al. [11] (2017)	Retrospective observational study	40	The safety and feasibility of using autologous and micro-fragmented adipose tissue.	12 months	No relevant complications nor clinical worsening were recorded.
Labarre et al. [12] (2022)	Prospective study	33	SVF treatment has the potential to improve the quality of life of patients by improving joint function and mobility.	1-year	No local adverse events were observed.
Lapuente et al. [13] (2020)	Retrospective and not controlled study	50	Intra-articular SVF infiltration for knee OA treatment is safe and effective.	1-year	There were no serious adverse effects.
Garza et al. [14] (2020)	Randomized controlled trial	39	Intra-articular SVF injections can significantly decrease knee OA symptoms and pain at 6 months and at 1 year.	6-month to 1-year	No adverse events.
Yokota et al. [15] (2019)	Cohort study	42	Both ASC and SVF resulted in clinical improvement in patients with knee OA.	6 months	No major complications occurred in either group.
Rothrauff et al. [16] (2019)	Controlled study	12	When compared with tears left untreated or repaired with suture alone, augmented repairs demonstrated increased tissue formation in the meniscal tear site, as seen on MRI and macroscopically. Likewise, the neotissue of augmented repairs possessed a histological appearance more similar, although still inferior to healthy meniscus.	6 months	No adverse events.
Koh et al. [17] (2015)	Prospective case series study	30	Almost all patients showed significant improvement in all clinical outcomes at the final follow-up examination.	2-year	No major complications associated with arthroscopic lavage and liposuction.
Nguyen et al. [18] (2017)	Experimental	30	AM with SVF/PRP injection was effective for knee OA and had better long-term outcomes than AM alone.	18 months	Included arterial hypertension, chest pain, dyspnea, and urinary retention.
Muñoz et al. [19] (2017)	Experimental	24	The supra- and infrapatellar fat pads of 24 patients with severe OA were resected during the surgical intervention for prosthetic implantation.	3 months	No severe adverse events.
Kim et al. [20] (2023)	Retrospective comparative study	156	Improved clinical and radiologic outcomes and favorable cartilage regeneration were seen after surgery for varus knee OA in both SVF and hucb-MSC groups.	33 months	Low complication.
Kim et al. [21] (2023)	Retrospective comparative study.	97	Significant correlations between the pain scores and MOCART scores were not observed in the SVF group until at the 6-month follow-up.	12 months	No severe adverse events.
Boada-Pladellorens et al. [22] (2022)	Retrospective	239	SVF was considered a safe treatment for knee OA and could hold promise in terms of pain, functionality, and improvement of anatomical structure.	6 to 24 months	Minor adverse effects.
Kim et al. [23] (2023)	Retrospective study	43	Showed an encouraging improvement in pain levels and cartilage regeneration after SVF implantation in patients with knee OA.	12 months	Low complication.
Yokota et al. [24] (2022)	Cohort study	80	ASC and SVF injections substantially improved knee pain and function.	24 months	No severe adverse events.
Aletto et al. [25] (2022)	Prospective study	123	Intra-articular knee injection of SVF is safe and effective to ameliorate the clinical and functional scores.	6 months	No complications were observed in the patients treated.
Santoprete et al. [26] (2022)	Retrospective	84	SVF injection into the knee joint of patients with OA resulted in symptomatic improvement at short-term follow-up.	7.9 months	The only complication noted was knee joint swelling lasting for less than 7 days after the injection in 7% of the patients.
Zhang et al. [27] (2022)	Retrospective	126	The cartilage volume was reduced in both the SVF and control groups at 5 years but reduced less in the SVF group.	5-year	No complications.
Simunec et al. [28] (2020)	Retrospective	12	Intra-articular injection of SVF is a safe and effective technique for the management of knee OA. Prior to an invasive artificial joint replacement, the treatment of arthritic knee joints with the intraarticular injection of autologous adipose tissue-derived SVF should be considered a regenerative treatment option.	12 months	No serious adverse events or unwanted side effects related to the SVF treatment were observed or reported.
Şahin et al. [29] (2021)	Experimental	18	Adipose-derived SVF improved healing of osteochondral defects treated with MF and HA-based scaffolds.		No complications.
Mehling et al. [30] (2020)	Retrospective	350	Subjects with stage III arthritis showed better results after SVF cell therapy.	3, 6, and 12 months	No complications.
Hudetz et al. [31] (2019)	Prospective, non-randomized study	20	Suggests that intra-articular injection of microfragmented adipose tissue decreases clinical symptoms in patients with late-stage knee OA, with no observed adverse events.	12 months	No complications.
Hong et al. [32] (2019)	Randomized Controlled Trial	16	Autologous treatment of SVF derived from adipose tissue is safe and can effectively relieve pain.	12-months	No severe adverse events.
Tran et al. [33] (2019)	Open-label, single-center, non-randomized, placebo-controlled, phase I/II clinical trial	33	SVF therapy is more effective in patients with KL grade 3 OA compared to patients with KL grade 2 OA.	24 months	No severe adverse events.

**Table 2:** SVF cell content isolated from the aqueous portion.

Type of Cells	Functions	Authors, Year [Ref.]
Mesenchymal Progenitor/Stem Cells	Capacity to perform self-renewal and differentiation into specific cell lineages and to support maintenance of other cells via paracrine secretion.	Spees et al., 2018 [36]
Lymphocytes	Participate in both innate and adaptive immune responses with multiple effect or functions. Produce antibodies, direct cell-mediated killing of virus-infected and/or tumor cells, and regulate immune responses.	Busato et al., 2020 [37]
Smooth Muscle Cells	Display involuntary contractile activity to control the diameter, wall movement, and wall stiffness of specific organs.	Guimarães, 2017 [38]
Adipose tissue-derived Stem Cells	Secrete growth factors, cytokines, and antioxidant factors into a microenvironment, regulating intracellular signaling pathways in neighboring cells. Protective outcome via inflammatory and immunomodulatory effects.	Bora et al., 2017 [34]
Preadipocytes	Promote growth of adipose tissue by differentiating into mature and metabolically active adipocytes. Proliferating preadipocytes may also exhibit phagocytic activity towards microorganisms and behave similarly to macrophage-like cells.	Matsuo et al., 2020 [39]
Mφ2 Macrophage	The type 2 macrophage (Mφ2) is produced by the type 2 T helper immune response and takes on an anti-inflammatory role, typically characterized by an increase in the production of interleukins (IL-4, IL-5, IL-9, and IL-13). It is also directly involved in regenerative and tissue repair processes that occur after injuries.	Contreras et al., 2015 [40]; Dey et al., 2021 [41]
T Cells	As components of the adaptive immune system with major importance, these cells are responsible for eliminating infected host cells, activating other immune cells and secreting cytokines that further regulate immune responses.	Dulong et al., 2022 [42]
Endothelial Precursor Cells and Endothelial Cells	Differentiate into functional endothelial cells and sustain vasculo genesis by incorporating themselves into the injured endothelium with the formation of functional blood vessels and through the local secretion of pro-angiogenic factors with a paracrine effect on the cells that form the vessel. Play a critical role in vascular homeostasis as well as physiological or pathological processes such as thrombosis, inflammation, and vascular wall remodeling. Resting endothelial cells control blood flow and the passage of protein from blood into tissues, as well as inhibiting inflammation and preventing coagulation.	Gulyaeva et al., 2019 [43]

**Table 3:** Effect of stromal vascular fraction on tissues.

Regulation of pro-inflammatory molecules	Decreases IL-1b and IL-6 levels. [39]
Hyaline cartilage extracellular matrix	Increases glycosaminoglycan level. [40]
Triggering of IL-1Ra	Reduces the catabolic effect of IL-1. [41]
Increasing of ADAMTS-4 and -5	Provides tissue balance (homeostasis). [43]
Anti-inflammatory	Reduces tissue swelling (edema). [44]
Anti-apoptotic	Reduces and stops programmed cell death. [44]
Increasing of TIMPs-1, -3, and -4 metalloproteinases	Provides tissue balance (homeostasis). [45]

ADAMTS: A Disintegrin and Metalloproteinase with Thrombospondin Motifs; TIMPs: Tissue Inhibitors of Metalloproteinases.

**Table 4:** Steps of stromal vascular fraction separation [46,47,48].

	Conventional	Modified Approach
Obtaining adipose tissue	- Abdominal fat. - Reusable Sorenson type lipoaspiration cannula. - Klein’s Translumination solution: Modified. - Klein solution (500 mL isotonic, 20 mL lidocaine, 2% epinephrine, 2 mL bicarbonate). - 50 mL Luer-Lock syringe	- Abdominal fat. - Disposable/Re-usable Coleman style cannula. - Klein’s Translumination solution: Modified. - Klein solution (500 mL isotonic, 20 mL lidocaine, 2% epinephrine, and 2 mL bicarbonate). - 50 mL Luer-Lock syringe.
Mechanical separation/shredding	- Shredding of tissue by shaking with glass ball (shaking time and strength depend on the user).	- Separation by the effect of gravity in a screw form mechanical separator at standard power and time.
Pre-filtration	- Polyethylene filtration in a 100 micrometer porous polyethylene bag.	- Filtration with the effect of gravity in the 100-micrometer porous device whose base will be supported by a metallic or polymeric cage.
Washing	[-]	- Washing in the device.
Final filtration	-Filtration on 10 micrometer porous polyethylene filters in 10 mL syringes.	- Final filtration with the rise of adipose tissue and SVF to the solution surface in serum within the device.
Collection of SVF/adipose tissue	- Available in an equivalent system.	- Proximal adipose tissue and SVF separation reservoir.
Cell counting and characterization	- Cell counting, determination of viability, determination of cell characteristics, and histochemical identification.	- Cell counting, determination of viability, determination of cell characteristics, and histochemical identification.

**Table 5:** Commercial medical products for AD-SVF preparation [47].

Product	Company	Article
Cha-Station	Somnotec http://www.somnotec.net	[53]
Octagone D200	Endecotts Ltd. https://www.endecotts.com	[54]
AdiPrep	Harvest http://www.harvest.co.kr/clinician/clinician-home/adiprep/advantages/quality.html	[55]
Lipokit	Medi-Khan http://www.medikanint.com	[56,57]
Puregraft 250	Puregraft LLC http://www.puregraft.com	[58]
Lipogems	Lipogems http://understandlipogems.com	[59,60]
MyStem	MyStem LLC https://mystem.eu/	[61,62]
Arthrex SVF	https://www.arthrex.com/orthobiologics	[63]
Adinizer	BSL http://biosl.com/?ckattempt=1	[64]
Microlyser	Tlab https://tlab.com.tr/en/products/microlyzer-svf-kit/	[65]
SEFFIE	Advanced-Maes http://www.advanced-maes.com/	[66]
LIPOCUBE	STEMC https://lipocube.com/	[67,68]
Q-Graft	Human Med AG https://www.humanmed.com/en/products/q-graft/	[28]
Tulip Nanotransfer	Tulip Medical https://tulipmedical.com/	[69]
Lipocell	Tissyou https://www.tissyou.com/portfolio_page/lipocell/	[70]
LipiVage	Genesis Biosystems https://www.genesisbiosystems.com/lipivagesystem-autologous-fat-transfer/	[71]

## Data Availability

Not applicable.
